# Pregnancy and appendicitis: a systematic review and meta-analysis on the clinical use of MRI in diagnosis of appendicitis in pregnant women

**DOI:** 10.1186/s13017-019-0254-1

**Published:** 2019-07-22

**Authors:** Mania Kave, Fateme Parooie, Morteza Salarzaei

**Affiliations:** 1 Gynecology and Obstetrics Institution, Faculty of Medicine, Zabol University of Medical Sciences, Zabol, Islamic Republic of Iran; 2 Student Research Committee, Faculty of Medicine, Zabol University of Medical Sciences, Zabol, Islamic Republic of Iran

**Keywords:** Pregnancy, Acute appendicitis, Magnetic resonance imaging (MRI)

## Abstract

**Background:**

The aim of this systematic review and meta-analysis was to evaluate the clinical use of MRI for the evaluation of acute appendicitis during pregnancy.

**Methods:**

The searches were conducted by two independent researchers (MK, MS) to find the relevant studies published from 1/1/2009 until end of 30/12/2018. We searched for published literature in the English language in MEDLINE via PubMed, EMBASETM via Ovid, The Cochrane Library, and Trip database. For literature published in other languages, we searched national databases (Magiran and SID), KoreaMed, and LILACS. The keywords used in the search strategy are Pregnancy [MeSH], Pregnant [MeSH] OR—Magnetic resonance imaging [MeSH] OR—Appendicitis [MeSH] OR—Ultrasound, [MeSH] OR, imaging, MRI [MeSH] OR"،" and Right lower quadrant pain [MeSH]. The risk of bias of every article was evaluated by using QUADAS-2. On the basis of the results from the 2 × 2 tables, pooled measures for sensitivity, specificity, diagnostic odds ratio (DOR), and area under the curves (AUC) along with their 95% confidence intervals (CIs) were calculated using the DerSimonian Lair methodology.

**Results:**

As many as 1164 studies were selected. After analyzing the correspondence of the studies with the required criteria, 19 studies were selected for the final review. For appendicitis in pregnancy, the MRI sensitivity was 91.8% at the 95% confidence interval of (95% CI 87.7–94.9%). At the confidence interval of 95%, the specificity was 97.9% (95% CI 0.97.2–100%). The risk of bias in the studies conducted was measured using the QUADAS-2 tool.

**Conclusion:**

MRI has high sensitivity and specificity (91.8%, 97.9% respectively) for the diagnosis of acute appendicitis in pregnant patients with clinically suspected appendicitis. It is an excellent imaging technique in many instances, which does not expose a fetus, or the mother, to ionizing radiation, making it an excellent option for pregnant patients with suspected acute appendicitis.

## Background

Approach to acute pain in the right lower quadrant in pregnancy is a challenge; It has various causes including digestive, gynecological and obstetrical, and renal causes. The possibility of acute appendicitis must be specifically ruled out, since it is the most common cause of surgical intervention in pregnancy requires immediate management [1–7]. The incidence rate of acute appendicitis in pregnancy has been reported to be 1:1250 and 1:1500 [8–15]. The possibility of acute appendicitis is higher in the second and third decades of life which are the fertility years [16–18]. Accurate diagnosis is difficult because the typical diagnostic clinical images are not present in all of the cases [19–25]. As the gestational age increases, the accuracy of the diagnosis decreases and the likelihood of appendical perforation and other complications will increase [26–29]. The negative laparotomy rate of suspected appendicitis is 25–50% in obstetric cases and 15–35% in general surgical cases [30–36]. Imaging in combination with physical examination will reduce the negative results of appendectomies [37–40]. Previous studies have indicated that CT (computed tomography) scan has better sensitivity and efficiency in comparison to US (ultrasound) [41–43]. Moreover, these studies have shown the high failure rate of US in diagnosing the disease even in patients whose appendicitis had been already confirmed by other imaging techniques [44–47]. The common usage of CT has been confirmed for evaluating patients suspected of appendicitis with reports on reduced healthcare costs for each patient and reduced rate of unnecessary appendectomy [48–52]. However, the fact that CT is applying ionizing radiation is worrying for both fetuses and pregnant women during the imaging process. Moreover, intravenous contrast is applied for increasing the diagnosis accuracy of CT, and it is associated with increased allergic reactions and contrast-inducted nephropathy. The changes made into the CT protocol can reduce exposure to fetal radiation less than 3 mGy, which is lower than the doses inducing adverse fetal effects [15, 53] mGy for the risk of carcinogenesis, 50 mGy for deterministic effects) [16, 54, 55]. However, CT is recommended in cases when inclusive clinical findings and ultrasound results are obtained or in situations in which MRI is not accessible. Thus, following the initial negative result of US, the American College of Radiology has introduced MRI (magnetic resonance imaging) as the recommended treatment [15, 16]. Therefore, the present systematic review and meta-analysis has been conducted to investigate the sensitivity,specificity, and diagnostic accuracy of MRI in diagnosing acute appendicitis in pregnant women.

## Methods

Presenting a systematic review and meta-analysis based on PRISMA [26] principles.

### Search methods for eligible studies

Searching for the eligible studies was conducted from 1/1/2009 to the end of 30/12/2018 by using the following searching strategy:

The searches were conducted by two independent researchers (MK, MS) to find the relevant studies published from 1/1/2009 to the end of 30/12/2018. We searched for published literature in the English language in MEDLINE via PubMed and EMBASETM via Ovid, The Cochrane Library, and Trip database. For literature published in other languages, we searched national databases (Magiran and SID), KoreaMed, and LILACS. To ensure literature saturation, the list of the included research references or the relevant reviews found by searching was studied (FP). The special search strategies were created using the Health Sciences Librarian website with specialization in systematic review searches using the MESH phrases and open phrases in accordance with the PRESS standards. After finalizing the MEDLINE strategy, the results were compared with searches from other databases (MS, FP). Similarly, PROSPERO was searched to find recent or ongoing systematic reviews. The keywords used in the search strategy were Pregnancy [MeSH], Pregnant [MeSH] OR—Magnetic resonance imaging [MeSH] OR—Appendicitis [MeSH] OR—Ultrasound, [MeSH] OR, imaging, MRI [MeSH] OR"،" , and Right lower quadrant pain [MeSH]. The list of previous study resources and systematic reviews were also searched for identifying the published studies (MK, MS). In addition, it was attempted to contact the authors of all studies that met the inclusion criteria and request unpublished data and abstracts (FP).

### Eligibility criteria

The inclusion criteria we used to select articles are as follows: (a) original retrospective and prospective blinded studies investigating the performance of MRI for appendicitis diagnosis in suspected pregnant women presenting with right lower quadrant pain; (b) using laparaoscopy open surgery or histopathologic examination as the standard reference, (c) containing a 2 × 2 table or included data that allowed the construction of a 2 × 2 table, (d) Described the diagnostic criteria for appendicitis on MRI in clear details, and (e) met quality standards, as assessed by the 14-item Quality Assessment of Diagnostic Accuracy Studies (QUADAS2) tool.

### Data extraction and risk of bias evaluation

The data were extracted for evaluating the characteristics of the participants. The index test included characteristics including special equipment, reference standard (executor of the tests and the interval between tests). The information related to diagnosis accuracy was also extracted. The first reader extracted the data (MS). The second reader confirmed the data (MK), and he would have completed them if they were incomplete.

The risk of bias of every article was evaluated using QUADAS-2 (a revised tool for quality assessment of diagnostic accuracy studies); four possible domains of bias results are evaluated. The first domain is patient selection (selecting the participants based on sequence or random). The participants of the present study are required to have the test conditions. Thus, the risk of bias is high in the studies; only participants suspected of appendicitis were selected. The second domain is the index test (wrong interpretation of the index test, accurate explanation of detection threshold). The third domain is the reference standard or “golden standard” (99% accuracy, the interpretation without considering the results of the index test). The last domain is the flow and timing (describing the patients receiving the index test, the time interval between index tests, and reference standard). Two reviewers evaluated the article independently with QUADAS-2 criteria (MS, FP). After independent evaluations, the reviewers discussed the article. Each domain was discussed to achieve a single view. The reliability of the reviewers for each domain was measured by using κ-statistic.

### Statistical analysis

On the basis of the results from the 2 × 2 tables, pooled measures for sensitivity, specificity, diagnostic odds ratio (DOR), and area under the curves (AUC) along with their 95% confidence intervals (CIs) were calculated using the DerSimonian Lair methodology [56]. Based on the pooled DOR of each index, test summary receiver–operator curves (sROC) were reconstructed using Moses–Shapiro–Littenberg methodology [57]. The DOR reflects the ability of a test to detect, in this case, appendicitis. A DOR of 1 indicates that the test has no discriminative power. The higher the DOR, the better the diagnostic ability of the imaging modality. To evaluate heterogeneity between studies, a Cochran Q statistic and the *I*^2^ index was used. A substantial *I*^2^ index indicates heterogeneity beyond sampling variation. A meta-regression analysis was performed to identify pre-defined sources of heterogeneity. We constructed the forest plots with the freeware Meta-DiSc, version 1.4, software (http://www.hrc.es/investigacion/ metadisc-en.htm; Ramon y Cajal Hospital; Madrid, Spain) [58]. The data related to the diagnostic accuracy of ultrasound were collected for providing a complete analysis. Then, for each of the categories, some studies were meta-analyzed; these studies had high and low risk of bias of participant selection (based on QUADAS-2 criteria). Sensitivity, specificity, and positive and negative likelihood ratios (LRs) were computed based on the true-positive, true-negative, false-positive, and false-negative rates for each study. Both LRs are independent from prevalence rates, and there is a consensus that a positive LR > 10 and a negative LR < 0.1 provide reliable evidence of satisfactory diagnostic performance [59]. The ratio of positive LR to negative LR was combined in a single global accuracy measure, the diagnostic odds ratio [60]. Summary sensitivity and specificity, positive and negative LRs, and diagnostic odds ratios were estimated by using a bivariate random effects model. This approach assumes bivariate normal distributions for the logit transformations of sensitivity and specificity from individual studies [61, 62]. In addition, the hierarchical summary receiver–operating characteristic (ROC) curve presenting the point estimates for each study, the joint ROC curve, and the pooled characteristics, including the 95% confidence region and the 95% prediction region, was constructed [63].

## Results

### Study selection

**Fig. 1:**
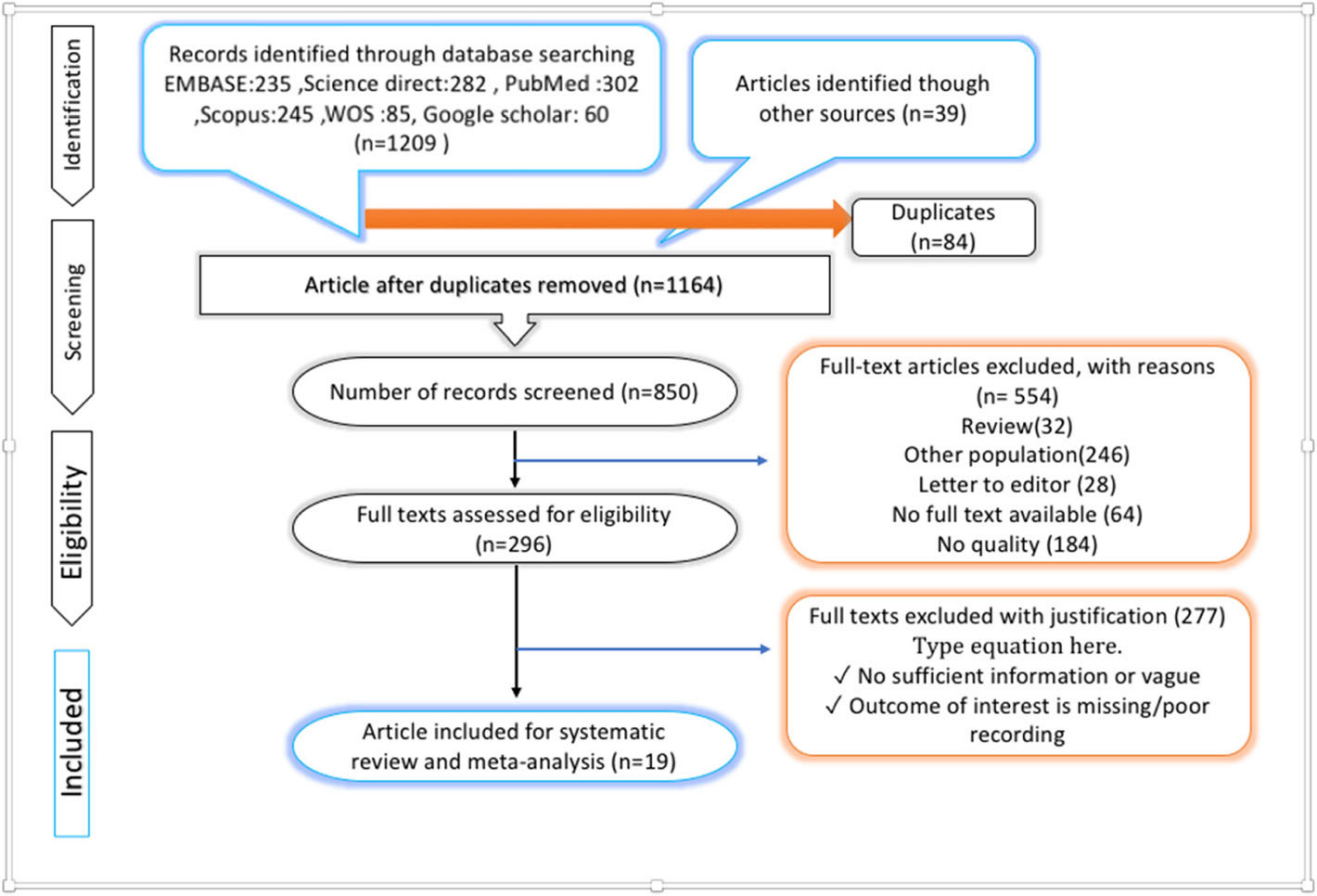
PRISMA flow diagram

Based on the searching strategy, as many as 1164 studies were selected. After analyzing the correspondence of the studies with the required criteria, 19 studies were selected for the final review (Fig. 1).

### Characteristics of the studies

The required characteristics of each selected study are indicated in Table 1. In total, 2400 patients (2400 pregnant women suspected of appendicitis presenting with right lower quadrant pain) were investigated in 19 studies. From these 19 studies, as many as 17 studies (94.7%) were retrospectives studies, and 2 studies (5.2%) were prospective studies. The investigated population were pregnant women suspected of appendicitis. The patients’ age ranged from 16 to 47 years. Seventeen studies applied 1.5-T MRI. Other studies have applied lower MRI field strength, and some have not reported it. From 2400 patients, 996 patients were evaluated with a magnetic strength of larger than 1 T. The MRI images obtained from all of the studies have been analyzed by an experienced radiologist which in most of the cases was a fellowship-trained attending radiologist.

**Table 1:** Summary of included studies

Author	Year	Country/province	Sampling method	Study design	Study duration	Setting	Mode of data collection	Participants	Age mean ± SD or range	Study population	Image interpretation
Theileu [64]	2014	USA	Convenience	Retrospective	2007–2012	Hospital	Interview	171	–	Pregnant women with suspected appendicitis	Board-certified attending
Ramaling am [65]	2015	USA	Convenience	Retropprospective	2007–2012	Hospital	Interview	102	16–41	Pregnant patients with acute abdominal pain	Fellowship radiologist trained in body MRI
Bichard [66]	2005	USA		Prospective	2002–2004	Hospital	Interview	29	–	Pregnant patients with acute abdominal pain	Fellowship radiologist trained in body MRI
Fonseca [67]	2014	USA	Convenience	Retrospective	2000–2011	Hospital	Interview	31	–	Pregnant women suspected for appendicitis	Attending radiologist
Isra [68]	2008	USA	Convenience	Retroprospective	2004–2006	Hospital	Interview	33	18–36	Pregnant women suspected for appendicitis	Attending radiologist
Rap [69]	2013	USA	Convenience	Retroprospective	1996–2011	Hospital	Interview	212	–	Pregnant patients with acute abdominal pain	Trained abdominal radiologist
Jang [70]	2011	South Korea	Convenience	Retroprospesctive	2008–2010	Hospital	Interview	18	–	Pregnant patients with acute appendicitis	Experienced gastrointestinal radiologist
Masselli [71]	2011	Italy	purposive	prospective	2006-2010	Hospital	Interview	40	20-35	pregnant patients with acute Abdominal and pelvic pain	Experienced radiologist in body MRI
Vu [72]	2009	Canada	Purposive	Retroprospective	2004–2008	Hospital	Interview	19	22–39	Pregnant patients with acute abdominal pain	Radiologist reponsible for reviewing abdominal MRI
Pedrosu [73]	2009	USA	Purposive	Retropresctive	2002–2007	Hospital	Medical records	148	15–42	Pregnant patients with acute abdominal pain	Fellowship trained attending radiologist
Oto [74]	2009	USA	Purposive	Retropresctive	2001–2007	Hospital	Hospital	118	18–40	Pregnant patients with acute abdominal and pelvic pain	Radiologist with subspecialty in abdominal MRI
Cobben [75]	2004	The Netherlands	Purposive	Presctive	2000–2003	Hospital	Interview	12	18–34	Pregnant patients with acute abdominal pain	Radiologist experienced in abdominal MRI
Aguilera [76]	2016	USA	Purposive	Retropresctive	2000–2011	Hospital	Interview	52	–	Pregnant patients with acute abdominal pain	Attending radiologist
Konrad [77]	2015	USA	Convenience	Retrospective	2009–2011	Hospital	Interview	140	–	Pregnant patients with suspected acute appendicitis	Attending radiologist
Sungah [78]	2016	South Korea	Convenience	Retrospective	2014-2016	Hospital	Interview	125	–	Pregnant patients with acute abdominal pain	Experienced radiologist
Kereshi [79]	2017	USA	Convenience	Retrospective	2010–2015	Hospital	Interview	204	17–47	Pregnant women with suspected appendicitis	Radiology fellow or abdominal imaging attend
Lauren M [80]	2015	USA	Convenience	Retrospective	2009–2014	Hospital	Interview	709	16–46	Pregnant patients with acute abdominal pain	Board-certified fellowship-trained abdominal radiologist
Richard [81]	2017	USA	Convenience	Retrospective	2007–2012	Hospital	Interview	223	–	Pregnant patients with acute abdominal pain	MRI fellows on any given day
Darshan [82]	2017	Canada	Purposive	Retrospective	2008–2015	Hospital	Interview	42	17–39	Pregnant patients with acute abdominal pain	Fellowship trained in body MRI

### Risk of bias

**Fig. 2:**
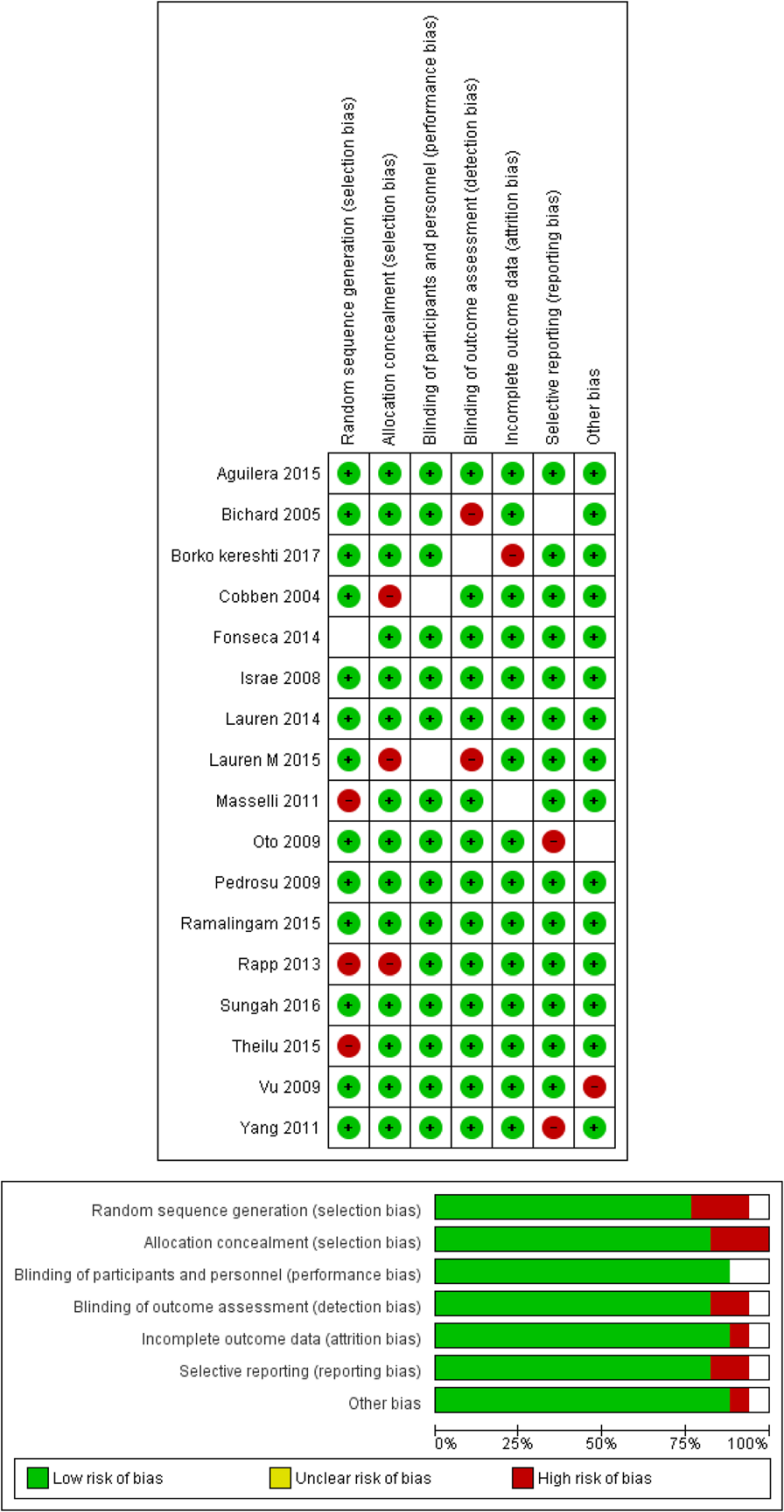
The risk of bias in the studies conducted was measured by using the QUADAS-2 tool. The risk of bias shown in Eq. 2 of the above image model (MRI) of each diagram indicate the number and percentage of studies with high (red), medium (yellow), and low (green) risk of bias in the four groups of the QUADAS-2 tool

**Fig. 3:**
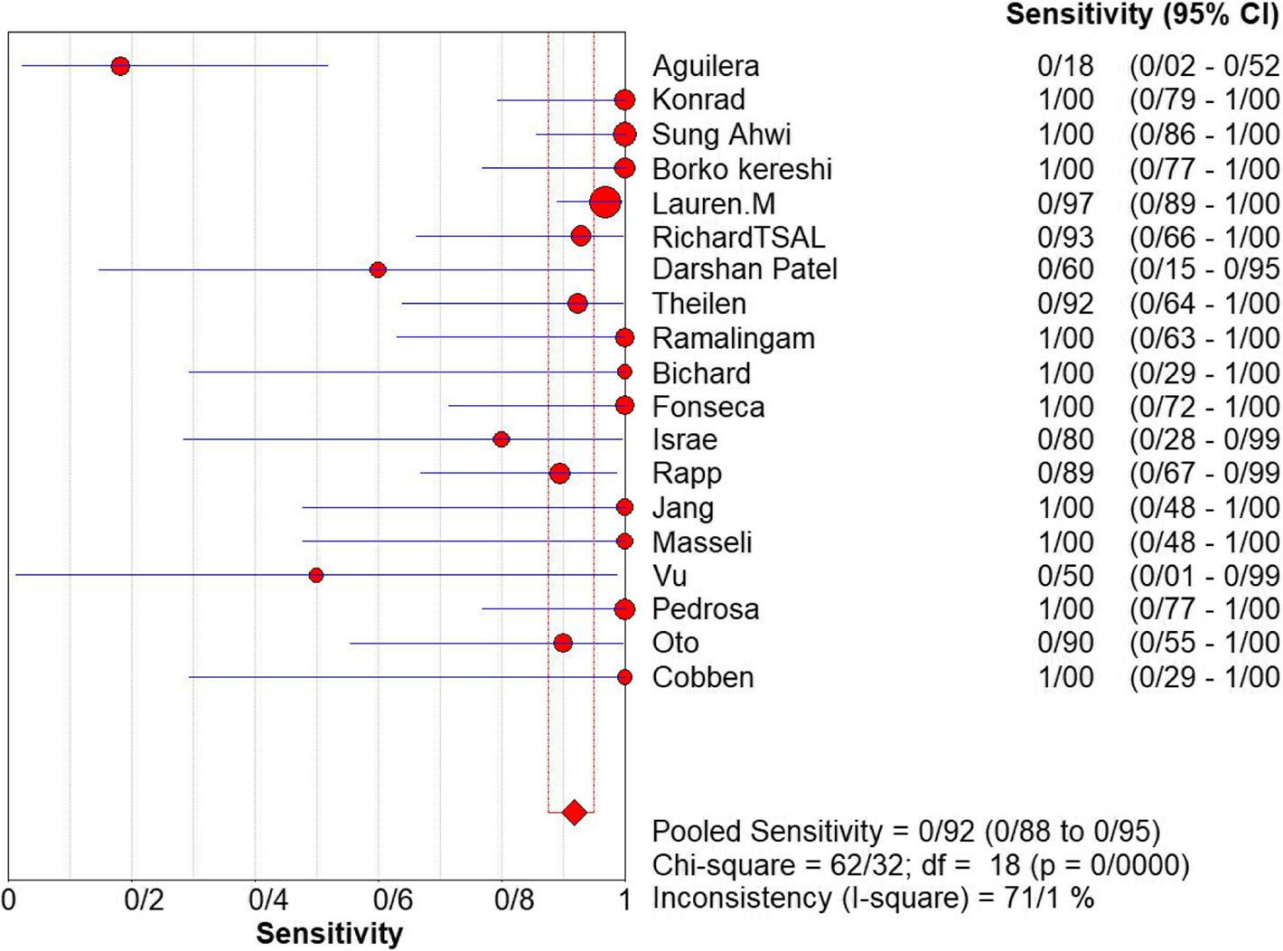
Sensitivity of MRI for diagnosing appendicitis in studies that included pregnant patients only. Forest plot of sensitivity reported in each study. Each study is identified by name of first author and year of publication, with circles representing individual study point estimates, size of each circle indicating relative contribution to data pooling (inverse variance weighting), horizontal lines indicating 95% CIs, and dashed vertical lines representing 95% CIs for pooled sensitivity and specificity

The findings of QUADAS-2 assessment have been indicated in Figs. 2 and 3; they indicate that only one parameter has a low risk of bias in the assessment. The studies have two or some specific limitations (Figs. 2 and 3).

### Overall meta-analysis

**Fig. 4:**
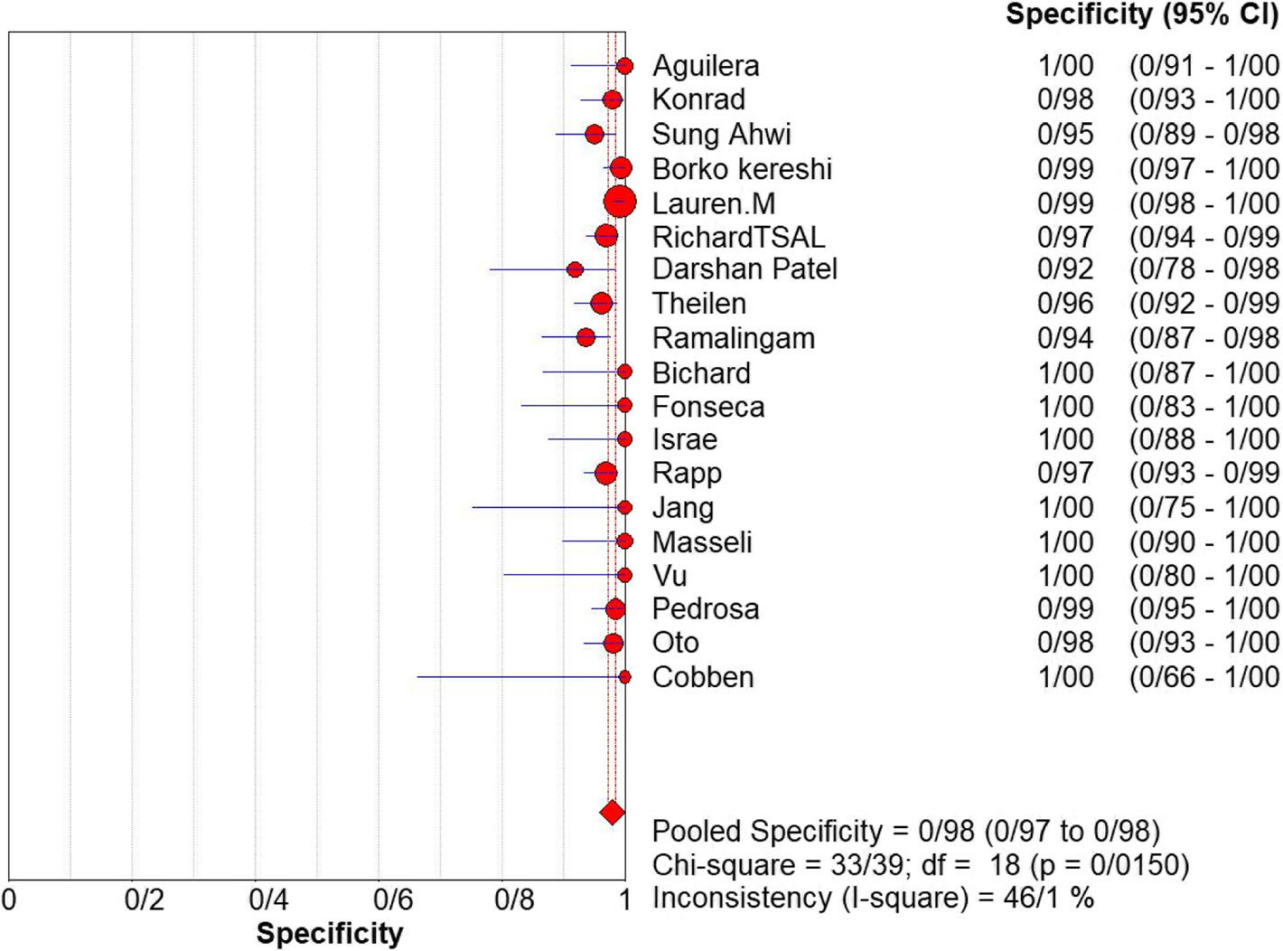
Specificity of MRI (≥ 1.5 T) for diagnosing appendicitis in studies that included pregnant patients only. Forest plots of specificity reported in each study. Each study is identified by name of first author and year of publication, with circles representing individual study point estimates, size of each circle indicating relative contribution to data pooling (inverse variance weighting), horizontal lines indicating 95% CIs, and dashed vertical lines representing 95% CIs for pooled specificity

**Fig. 5:**
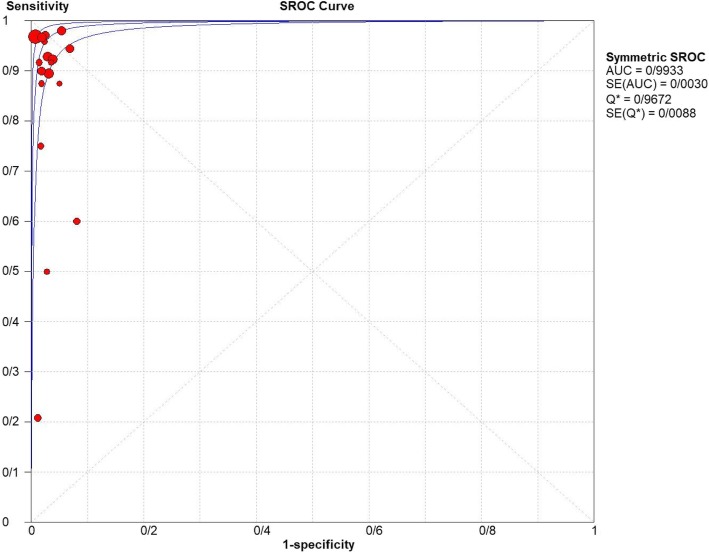
Summary-ROC (SROC) curve for diagnostic accuracy of MRI in diagnosing appendicitis. Size of each circle on graph represents sample size of included study. SE = standard error; Q* index = point at which sensitivity and specificity are equal or point closest to ideal top-left corner of SROC space

**Fig. 6:**
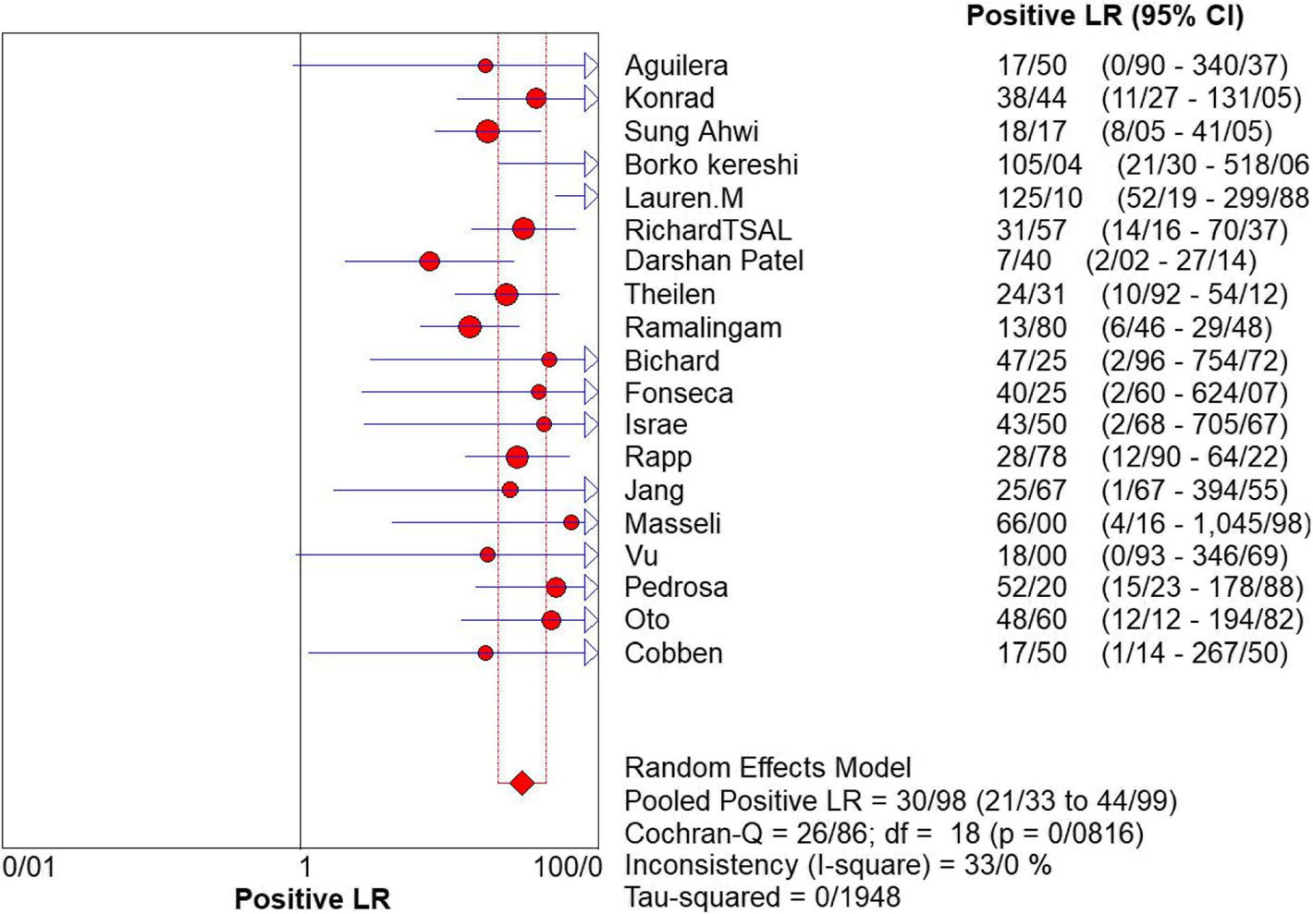
Positive LR of MRI for diagnosing appendicitis in studies that included pregnant patients only. Forest plot of positive LR reported in each study. Each study is identified by name of first author and year of publication, with circles representing individual study point estimates, size of each circle indicating relative contribution to data pooling (inverse variance weighting), horizontal lines indicating 95% CIs, and dashed vertical lines representing 95% CIs for pooled positive LR

**Fig. 7:**
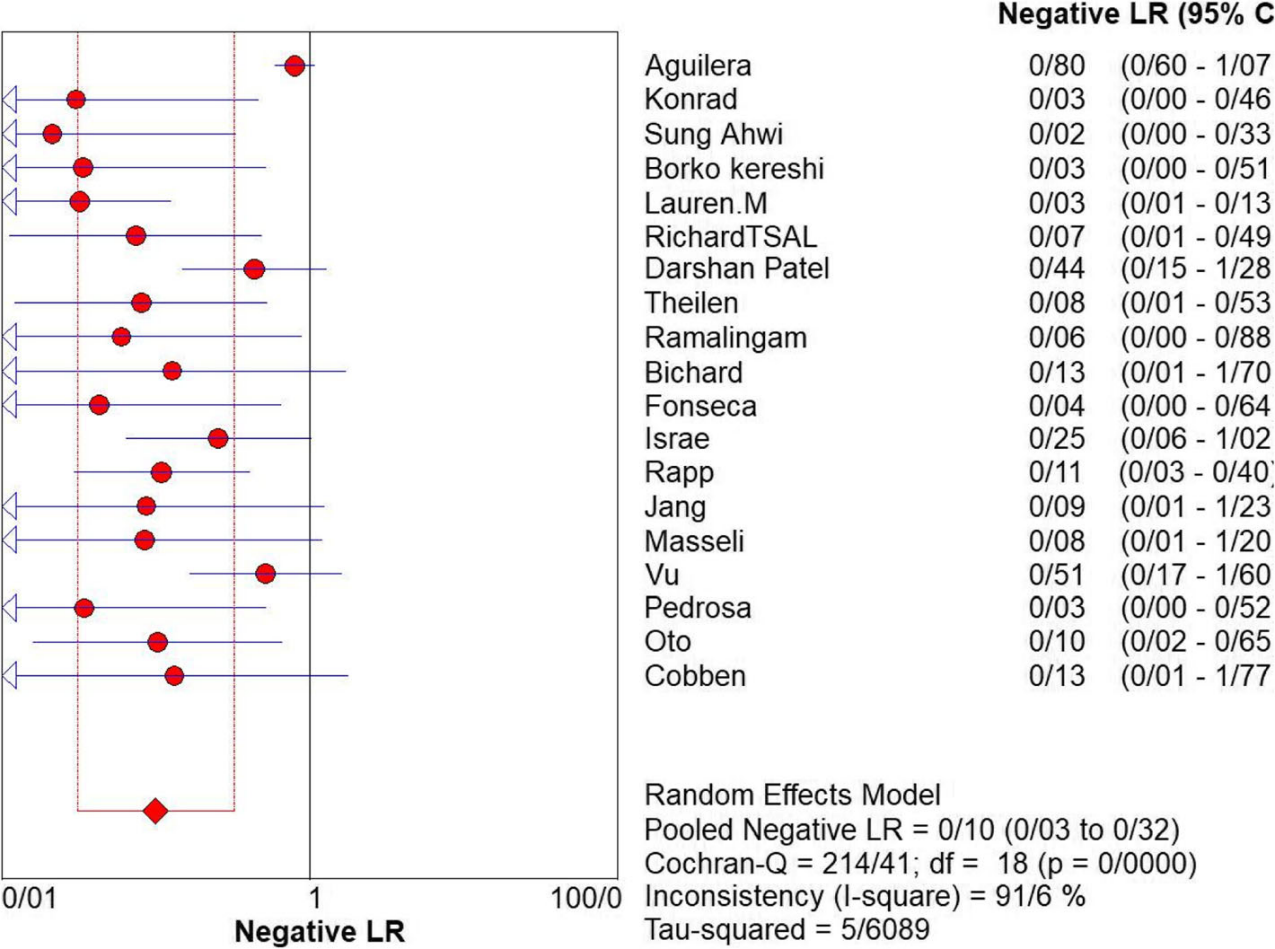
Negative LR of MRI for diagnosing appendicitis in studies that included pregnant patients only. Forest plot of negative LR reported in each study. Each study is identified by name of first author and year of publication, with circles representing individual study point estimates, size of each circle indicating relative contribution to data pooling (inverse variance weighting), horizontal lines indicating 95% CIs, and dashed vertical lines representing 95% CIs for pooled Negative LR

For appendicitis in pregnancy, the MRI sensitivity was 91.8% at the confidence interval of 95 percent (95% CI 87.7–94.9%). At the confidence interval of 95%, the specificity was 97.9% (95% CI 97.2–100%). The diagnostic odds ratio was 177.60 (95% CI 35.012–900.91) showing a high accuracy of MRI in diagnosing appendicitis in pregnant women (Table 2). The SROC plot showed a summary of estimated sensitivity and specificity and the area under the SROC curve of MRI in diagnosing appendicitis in pregnant women (Figs. 3, 4, and 5). The positive and negative LRs for MRI in diagnosing appendicitis in pregnant women were 30.98(21.33 to 44.99) and 0.10(0.03 to 0.32) respectively (Figs. 6 and 7).

**Table 2:** Accuracy of individual studies MRI in characterization of acute appendicitis during pregnancy

Study	Year	Participants	TP	FP	FN	TN	Sensitivity(95% CI)			Specificity(95% CI)			Accuracy
							95%	Low	Up	95%	Low	Up	
Theilen	2014	171	12	6	1	152	0.923	0.640	0.998	0.962	0.919	0.986	92%
Ramalingam	2015	102	8	6	0	88	1.000	0.631	1.000	0.936	0.866	0.976	100%
Bichard	2005	29	3	0	0	26	1.000	0.292	1.000	1.000	0.868	1.000	100%
Fonseca	2014	31	11	0	0	20	1.000	0.715	1.000	1.000	0.832	1.000	100%
Isra	2008	33	4	0	1	28	0.800	0.284	0.995	1.000	0.877	1.000	80%
Rap	2013	212	17	6	2	187	0.895	0.669	0.987	0.969	0.934	0.989	89%
Jang	2011	18	5	0	0	13	1.000	0.478	1.000	1.000	0.753	1.000	100%
Masselli	2011	40	5	0	0	35	1.000	0.478	1.000	1.000	0.753	1.000	100%
Vu	2009	19	1	0	1	17	0.500	0.013	0.987	1.000	0.805	1.000	50%
Pedrosu	2009	148	14	2	0	132	1.000	0.768	1.000	0.985	0.947	0.998	100%
Ato	2009	118	9	2	1	106	0.900	0.555	0.997	0.981	0.935	1.000	50%
Cobben	2004	12	3	0	0	9	1.000	0.292	1.000	1.000	0.664	1.000	100%
Aguilera	2016	52	2	0	9	41	0.182	0.023	0.518	1.000	0.914	1.000	18%
Konrad	2015	140	16	2	0	96	1.000	0.794	1.000	0.980	0.928	0.998	100%
Sungah	2016	125	24	5	0	96	1.000	0.858	1.000	0.950	0.888	0.984	100%
Borkokereshi	2017	176	14	1	0	161	1.000	0.768	1.000	0.994	0.982	1.000	100%
Lauren M	2015	709	61	5	2	641	0.968	0.890	0.996	0.992	0.982	0.997	96%
Richard	2017	223	13	6	1	198	0.929	0.661	0.998	0.971	0.937	0.989	92%
Darshan	2017	42	3	3	2	34	0.600	0.147	0.947	0.919	0.781	0.983	60%
Pooled sensitivity and specificity	–	2400	135	44	110	2080	0.918	0.877	0.949	0.979	0.972	1.000	–

*TP*, true positive; *FP*, false positive; *FN*, false negative; *TN*, true negative; *95% CI*, 95% confidence interval

### Meta-regression

Meta-regression indicated that the mean field strength of MRI was intervening (*p* = 0.0017) (Table 3).

**Table 3:** The quality of the articles that is calculated using a checklist which includes 5 criteria

First Author	Country	Year	Tesla	Sample size	Mean age	Accuracy	NPV	PPV
Birchard	USA	2005	1.5	√	√	*	*	*
Cobben	-	2004	1	√	√	*	*	*
Fonseca	USA	2014	N/A	√	*	*	*	*
Israel	USA	2008	1.5	√	√	√	√	√
Jang	Korea	2011	1.5	√	√	*	*	*
Masselli	Italy	2009	1.5	√	*	*	√	√
Pedrosa	USA	2009	1.5	√	√	*	√	√
Ramalingam	USA	2014	1.5	√	√	*	√	√
Rapp	USA	2013	1.5	√	√	*	√	√
Theilen	USA	2014	1.5	√	√	*	√	√
Vu	Canada	2009	1.5	√	√	√	√	√
Oto	USA	2008	1.5	√	√	√	√	√
Patel	Canada	2017	1.5	√	√	√	√	√
Kereshi	USA	2017	1.5	√	√	*	√	√
Ah Wi	Korea	2018	1.5	√	√	√	√	√
Burke	USA	2015	1.5	√	√	√	√	√
Konrad	USA	2015	1.5	√	*	*	√	√
Aguilera	USA	2018	1.5	√	√	*	√	√
Theilen	USA	2014	1.5	√	*	*	√	√

Based on these 5 criteria, articles were scored and then classified to three different quality including good quality (score more than 4), average quality (score 3–4), and weak quality (score below 3). Six studies had good quality

### Subgroup analysis of MRI field of strength

**Fig. 8:**
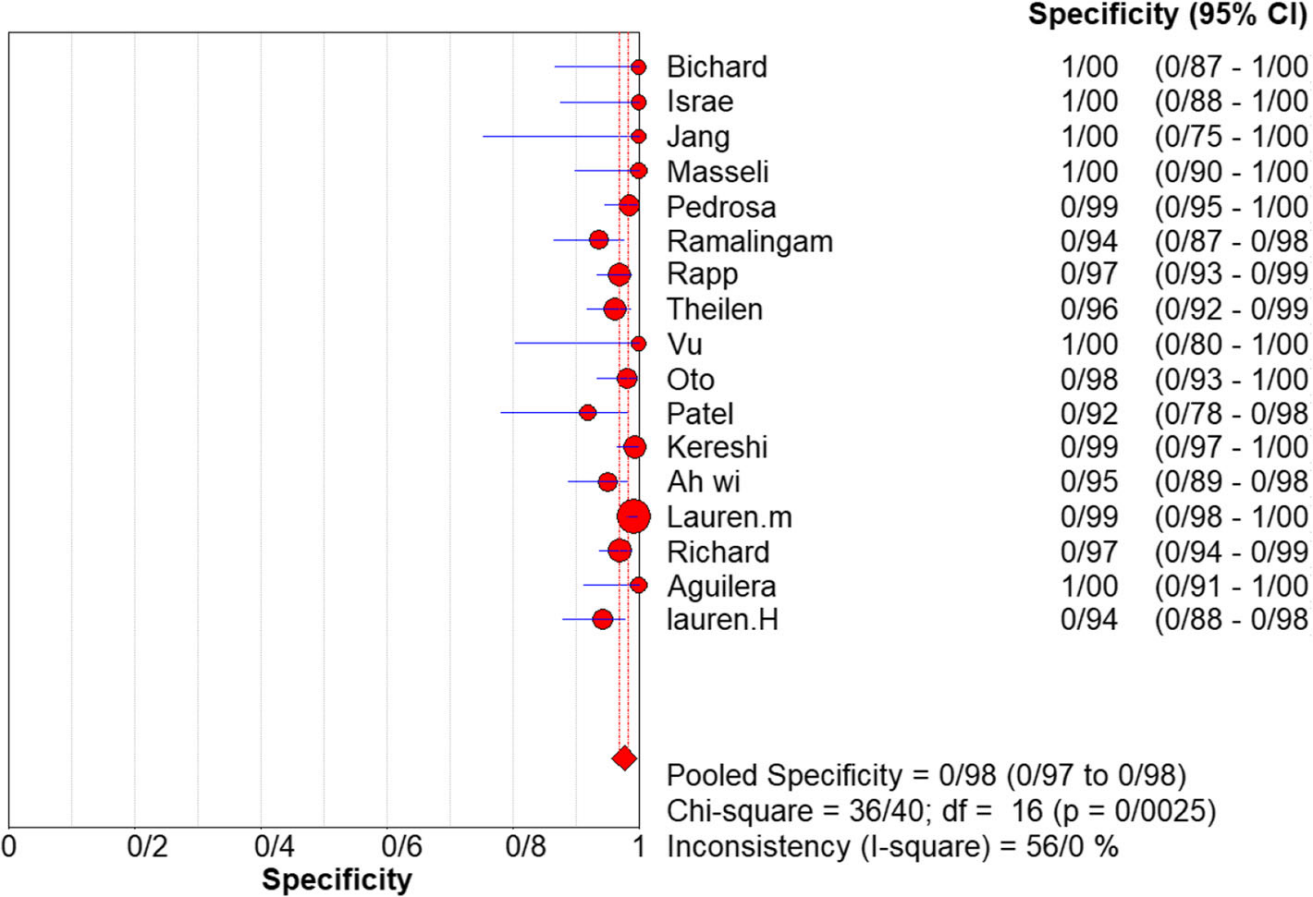
Specifity of MRI (≥ 1.5 T) for diagnosing appendicitis in studies that included pregnant patients only. Forest plot of sensitivity reported in each study. Each study is identified by name of first author and year of publication, with circles representing individual study point estimates, size of each circle indicating relative contribution to data pooling (inverse variance weighting), horizontal lines indicating 95% CIs, and dashed vertical lines representing 95% CIs for pooled sensitivity and specificity

Analyzing studies with the field strength of ≥ 1.5 T indicated that sensitivity was 0.94% (95% CI 88–0.98%) and specificity was 0.92% (at the confidence interval of 95% CI 0.64–100%). DOR was 325.74 (at the confidence interval of 95%) showing a very high accuracy of MRI with field strength of ≥ 1.5 T in diagnosing appendicitis in pregnant women. and inconsistency was 56%. The SROC plot. The SROC plot showed a summary of estimated sensitivity and specificity and the area under the SROC curve of MRI with a field strength of ≥ 1.5 T in diagnosing appendicitis in pregnant women (Figs. 6, 7, and 8).

### Country

**Fig. 9:**
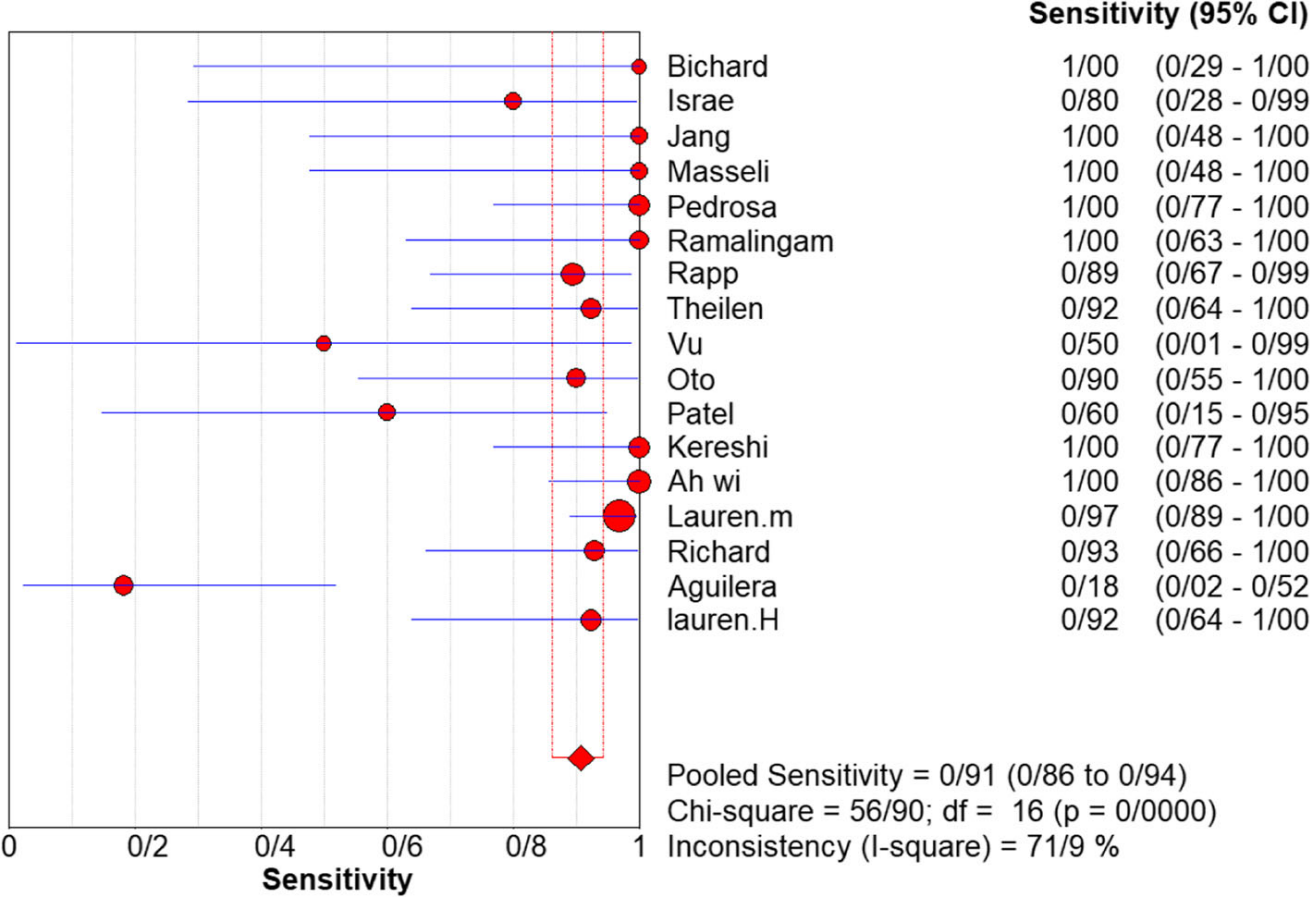
Sensivity of MRI (≥ 1.5 T) for diagnosing appendicitis in studies that included pregnant patients only. Forest plots of specificity reported in each study. Each study is identified by name of first author and year of publication, with circles representing individual study point estimates, size of each circle indicating relative contribution to data pooling (inverse variance weighting), horizontal lines indicating 95% CIs, and dashed vertical lines representing 95% CIs for pooled specificity

**Fig. 10:**
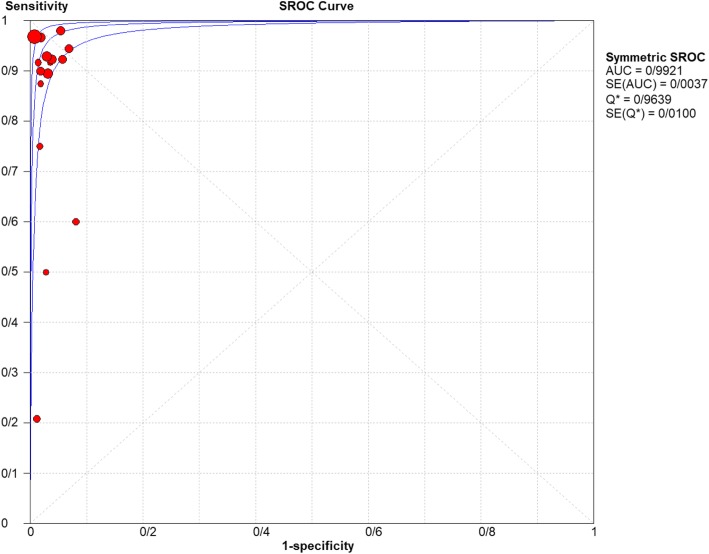
Summary-ROC (SROC) curve for diagnostic accuracy of MRI (≥ 1.5 T) in diagnosing appendicitis. Size of each circle on graph represents sample size of included study. SE = standard error; Q* index = point at which sensitivity and specificity are equal or point closest to ideal top-left corner of SROC space

**Fig. 11:**
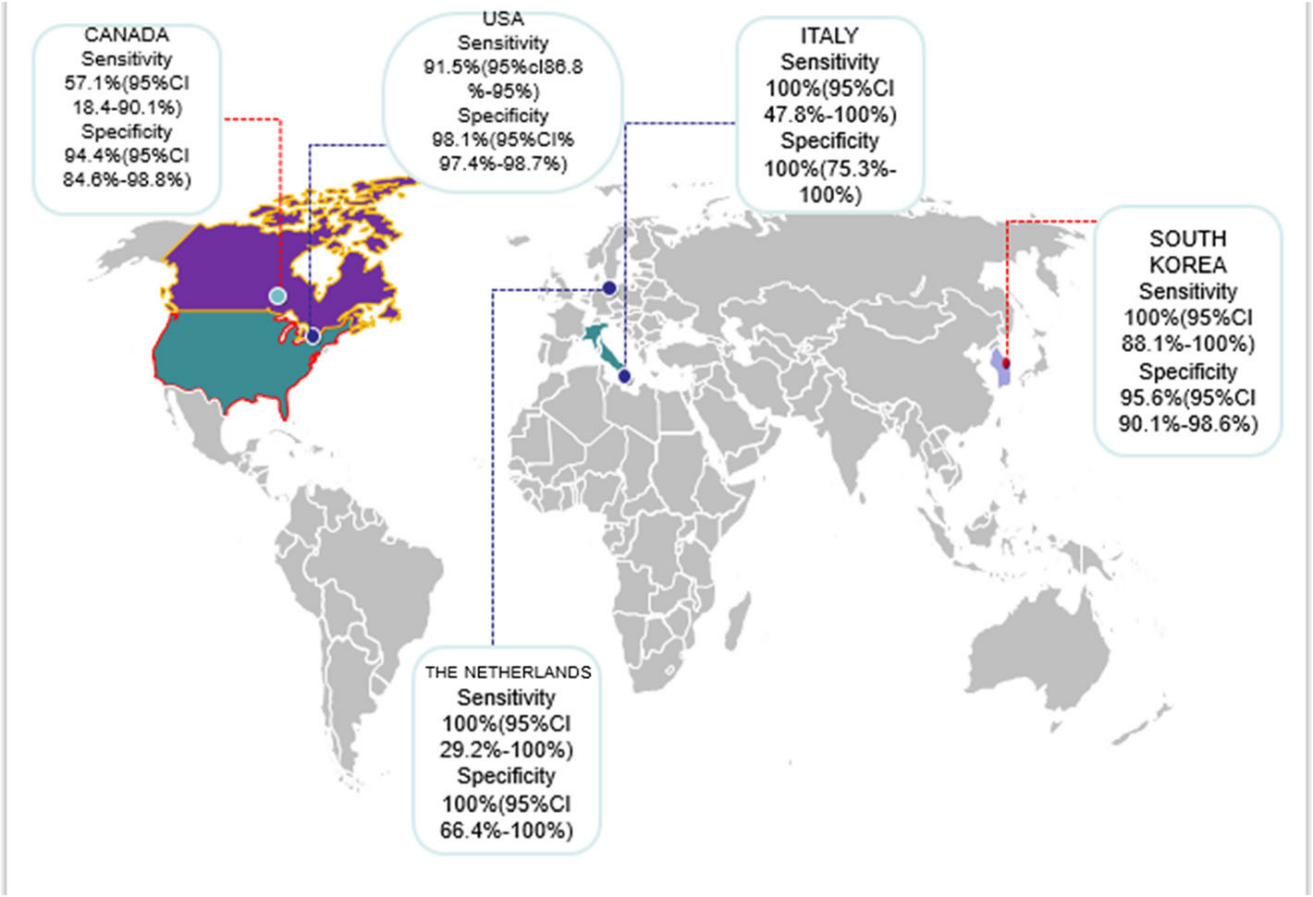
Pooled sensitivity and specificity of MRI in diagnosis of appendicitis in pregnancy by countries

Analyzing 13 studies conducted in USA [64–69, 73, 74, 76, 77, 79–81] indicated a pooled sensitivity of 91.5% (95% CI 86.8–95%) and a pooled specificity of 98.1%(95% CI 97.4–98.7%) and a DOR of 559.41 (95% CI 262.40–1192.6) (Figs. 9 and 10). the sensitivity, specificity, and DOR of MRI in diagnosis of appendicitis in pregnant women in South Korea based on 2 included articles [70, 78] were 100% (95% CI 88.1–100%), 95.6% (95% CI 90.1–98.6%), 596.36 (95% CI 55.640–6391.9) respectively. The sensitivity, specificity, and DOR of MRI in diagnosis of appendicitis in pregnant women in Canada based on 2 included articles [72, 82] were 57.1% (95% CI 18.4–90.1%), 94.4% (95% CI 84.6–98.8%), 20.523 (95% CI 3.250–129.61) respectively (Fig. 11).

## Discussion

Many investigators have previously shown that MRI for the workup of acute appendicitis in pregnancy is highly reliable and useful. In this systematic review and meta-analysis, we calculated a 91.8% sensitivity and a 97.9% specificity of MRI for the diagnosis of appendicitis in pregnant women. We were able to include 7 more studies than the most recent published meta-analysis by Eugene Duke et al [83] included, which reviewed 12 studies between 2004 and 2015 comprising 933 pregnant women and calculated a pooled sensitivity of 94% (95% CI 87–98%), and specificity of 97% (95% CI 96–98%) and a DOR of 309.8 (95% CI 140.5–711). Also, analyzing studies with the field strength of ≥ 1.5 T (which was the field strength used in most of the studies included) indicated that sensitivity was 0.94% (95% CI 88–0.98%) and specificity was 0.92% (at the confidence interval of 95% CI 0.64–100%). DOR was 325.74 (at the confidence interval of 95%) which is indicative of the better accuracy of MRI with a field strength of ≥ 1.5 T in diagnosing appendicitis in pregnant women. Our calculated sensitivity and specificity was lower than what Blumenfeld YJ et al. [84] reported in their meta-analysis (specificity of 99.9% and sensitivity of 95.0%). Due to the high number of included studies which were conducted in the USA [64–69, 73, 74, 76, 77, 79–81], we decided to perform a subgroup meta-analysis based on the country which was indicative of a pooled sensitivity of 91.5% (95% CI 86.8–95%) and a pooled specificity of 98.1% (95% CI 97.4–98.7%) and a DOR of 559.41 (95% CI 262.40–1192.6) for MRI in diagnosis of appendicitis in pregnant women. The sensitivity, specificity, and DOR of MRI in diagnosis of appendicitis in pregnant women in South Korea based on 2 included articles [70, 78] were 100% (95% CI 88.1–100%), 95.6% (95% CI 90.1–98.6%), and 596.36 (95% CI 55.640–6391.9) respectively. Mahesh K et al. [85] in a 5-year study on 39 pregnant women reported that the sensitivity of CT in the diagnosis of appendicitis in pregnant women was 100%; they also reported a sensitivity of 46.1% for ultrasound, but on the other hand, Kevin A et al. [86] in their meta-analysis presented a sensitivity of 89.9% and a specificity of 93.6% for CT scan in diagnosing the acute appendicitis in adults including pregnant women which is quite close and even in some countries like South Korea, The Netherlands, and Italy, less than what we calculated as the sensitivity and specificity of MRI in diagnosing acute appendicitis in pregnant women. The SAGES guidelines do not recommend the employment of CT scan as the initial imaging technique for pregnant patients, except in cases where urgent information is needed for trauma or acute abdominal pain [87–90]. Given the effectiveness of ultrasound and MRI, CT should be used only in emergency cases or in situations where MRI is inaccessible or cannot be used [91, 92]. When ionizing radiation imaging is required, specific techniques can be employed in accordance with as low as reasonably achievable (ALARA) principle [93]. MR imaging can be performed without using intravenous gadolinium for pregnant women. MRI is favored compared with CT scan to diagnose non-obstetric abdominal pain in gravid patients, as the former makes it possible to take excellent soft tissue images without using ionizing radiation and is safer when applied to pregnant patients [67, 94, 95].

The maternal and fetal outcomes can be improved if abdominal conditions during pregnancy are diagnosed accurately and timely. Diagnostic laparoscopy is a preferred choice for cases where available resources prevent prompt imaging for diagnosis or when imaging is inconclusive. The risks of delayed diagnosis should be compared with possible risks associated with possible negative laparoscopy. The conditions diagnosed at laparoscopy should be treated by the surgeon as soon as possible [96].

The results of this study indicate that although there is a small difference between CT scan and MRI sensitivity in diagnosing appendicitis in pregnancy due to multiple complications of CT scan in pregnancy including exposure of patients to ionizing radiation, which is of special concern in pediatric and obstetric populations [87], MRI seems to be a more reasonable imaging modality than CT scan in cases of suspected appendicitis in pregnancy, especially in tertiary care centers that have access to specialized radiologists.

Limitations of this meta-analysis mostly relate to the available data and the heterogeneity of design, interpretation of results, and reporting of data in primary studies. Our study is also limited by the fact that most of the studies were retrospective case series. The number of patients enrolled ranged from 12 to 709, which in some cases led to inconsistencies in the results, especially in the calculated accuracies of MRI. and most studies did not include the overall appendicitis rates in their respective institutions, and thus, we were unable to calculate prevalence-adjusted positive and negative predictive values. Also, the studies varied by their inclusion criteria.

## Conclusion

MRI has high sensitivity and specificity (91.8% and 97.9% respectively) for the diagnosis of acute appendicitis in pregnant patients with clinically suspected appendicitis. It is an excellent imaging technique in many instances, which does not expose a fetus, or the mother, to ionizing radiation, making it an excellent option for pregnant patients with suspected acute appendicitis. It can be performed at any stage of pregnancy, with no evidence of adverse effects on fetal outcomes, as it is currently being used. As radiologists become increasingly comfortable with interpreting abdominal and pelvic MRI, and as it becomes more widely available as an emergent procedure, its utility will continue to increase in the future.

## Data Availability

Please contact the authors for data requests.

## Abbreviations

CT: Computed tomography
DOR: Diagnostic odds ratio
LR: Likelihood ratio
MRI: Magnetic resonance imaging
SROC: Summary receiver–operator curves
US: Ultrasound
